# Mechanical interactions between the biceps femoris long and short heads: Implications for T‐junction hamstring injuries

**DOI:** 10.1111/cpf.70026

**Published:** 2025-08-28

**Authors:** Gakuto Nakao, Ginji Nara, Risa Adachi, Koki Ishiyama, Kazuyoshi Kozawa, Keita Sekiguchi, Kanna Nagaishi, Kousuke Shiwaku, Norio Hayashi, Jurdan Mendiguchia, Raki Kawama, Nobuhiro Aoki, Masaki Katayose, Keigo Taniguchi

**Affiliations:** ^1^ Graduate School of Health Sciences Sapporo Medical University Sapporo Japan; ^2^ Sapporo Medical Technology, Professional Postsecondary Course (Physical Therapist) Welfare and Dentistry Professional Training College of Nishino Gakuen School Foundation Sapporo Japan; ^3^ Rehabilitation Center NTT Medical Center Sapporo Sapporo Japan; ^4^ Department of Anatomy, Division of Functional Morphology, School of Medicine Sapporo Medical University Sapporo Japan; ^5^ Department of Orthopaedic Surgery, School of Medicine Sapporo Medical University Sapporo Japan; ^6^ Musculoskeletal Functional Anatomy Research Institute Gifu Japan; ^7^ Department of Physical Therapy ZENTRUM Rehab and Performance Center Barañain Spain; ^8^ Faculty of Health and Sports Science Doshisha University Kyoto Japan; ^9^ Organization for Research Initiatives and Development Doshisha University Kyoto Japan; ^10^ Department of Physical Therapy, School of Health Sciences Sapporo Medical University Sapporo Japan

**Keywords:** biceps femoris short head, force transmission, hamstring mechanics, intermuscular connections, shear wave elastography

## Abstract

Although force transfer during elongation occurs longitudinally and transversely, the influence of transverse force transfer between the biceps femoris long head and short head remains unclear. This study aimed to investigate whether separating the intermuscular connections between the biceps femoris long head and short head alters tension in the biceps femoris long head. Eight human cadaver legs were used, and ultrasonic shear wave elastography measurements were performed under four conditions: (1) intact, (2) removal of all tissues from the skin to the deep fascia, (3) intermuscular dissection, and (4) biceps femoris short head detachment. Measurements were taken in four limb positions, defined by hip and knee joint angles, under each tissue condition. The shear modulus of the biceps femoris long head significantly increased by 62.2% after intermuscular dissection compared to fascia removal, and further increased by 174.7% after biceps femoris short head detachment. In contrast, the shear modulus of the biceps femoris short head significantly decreased by 36.0% following intermuscular dissection and by 75.1% after detachment. In conclusion, reducing biceps femoris short head tension while increasing biceps femoris long head tension may influence muscle stress distribution, particularly during movement.

## 1 INTRODUCTION

The biceps femoris muscle comprises two distinct heads: the long head (BFlh) and the short head (BFsh). The BFlh originates from the ischial tuberosity and spans the hip and knee joints, whereas the BFsh originates from the femur and crosses only the knee joint. A notable anatomical feature of the biceps femoris is the distal musculotendinous junction, commonly referred to as the T‐junction, where the epimysial surfaces of the BFlh and BFsh converge (Entwisle et al., 2017). This region is the most frequent site of severe hamstring injuries and is associated with a reinjury rate of up to 54%, significantly higher than the general hamstring reinjury rate (Entwisle et al., 2017; Kenneally‐Dabrowski et al., 2019; Shamji et al., 2021). Injuries involving the T‐junction have also been linked to prolonged recovery and an increased risk of recurrence (Entwisle et al., 2017; Shamji et al., 2021). However, recent evidence suggests that T‐junction involvement does not necessarily result in worse clinical outcomes in terms of time to return to sport or reinjury rates (Cronin et al., 2024; Kerin et al., 2024). Clarifying the mechanical roles of the BFlh and BFsh, particularly at this junction, may provide important insights for injury prevention and rehabilitation.

Recent research has proposed a potential mechanical interaction between the BFlh and BFsh because of their shared distal tendon. Nakao et al. used ultrasonic shear wave elastography (SWE) and demonstrated that detachment of the BFsh origin from the femur resulted in an increase in the shear modulus of the BFlh (Nakao, Yamagata, et al., 2025), suggesting that the BFsh may contribute to modulation of BFlh tension. However, force transmission during elongation occurs along the longitudinal and transverse axes (Finni et al., 2023). Regarding morphological features of the hamstrings, Farfán et al. reported that the semitendinosus muscle often partially originates from the BFlh via a shared fibrous membrane (Farfán et al., 2021), suggesting a structural basis for transverse or lateral force transmission between adjacent hamstring muscles. These findings imply that the architecture and functional behavior of the hamstrings cannot be fully explained by longitudinal mechanics alone, highlighting the importance of intermuscular connections in muscle coordination and tension distribution. Given the anatomical structure of the musculotendinous T‐junction of the biceps femoris, the tension generated within each head may be distributed to adjacent muscles through intermuscular connections (Entwisle et al., 2017). However, previous studies have only examined BFsh origin detachment from the femur, without accounting for potential transverse force transmission (Nakao, Yamagata, et al., 2025), leaving it unclear how separating these connections affects BFlh tension. Therefore, this study aimed to determine whether separating the intermuscular connections between the BFsh and BFlh alters BFlh tension. We hypothesized that this separation would increase BFlh tension.

## 2 METHODS

### 2.1 Experimental protocol

**Figure 1:**
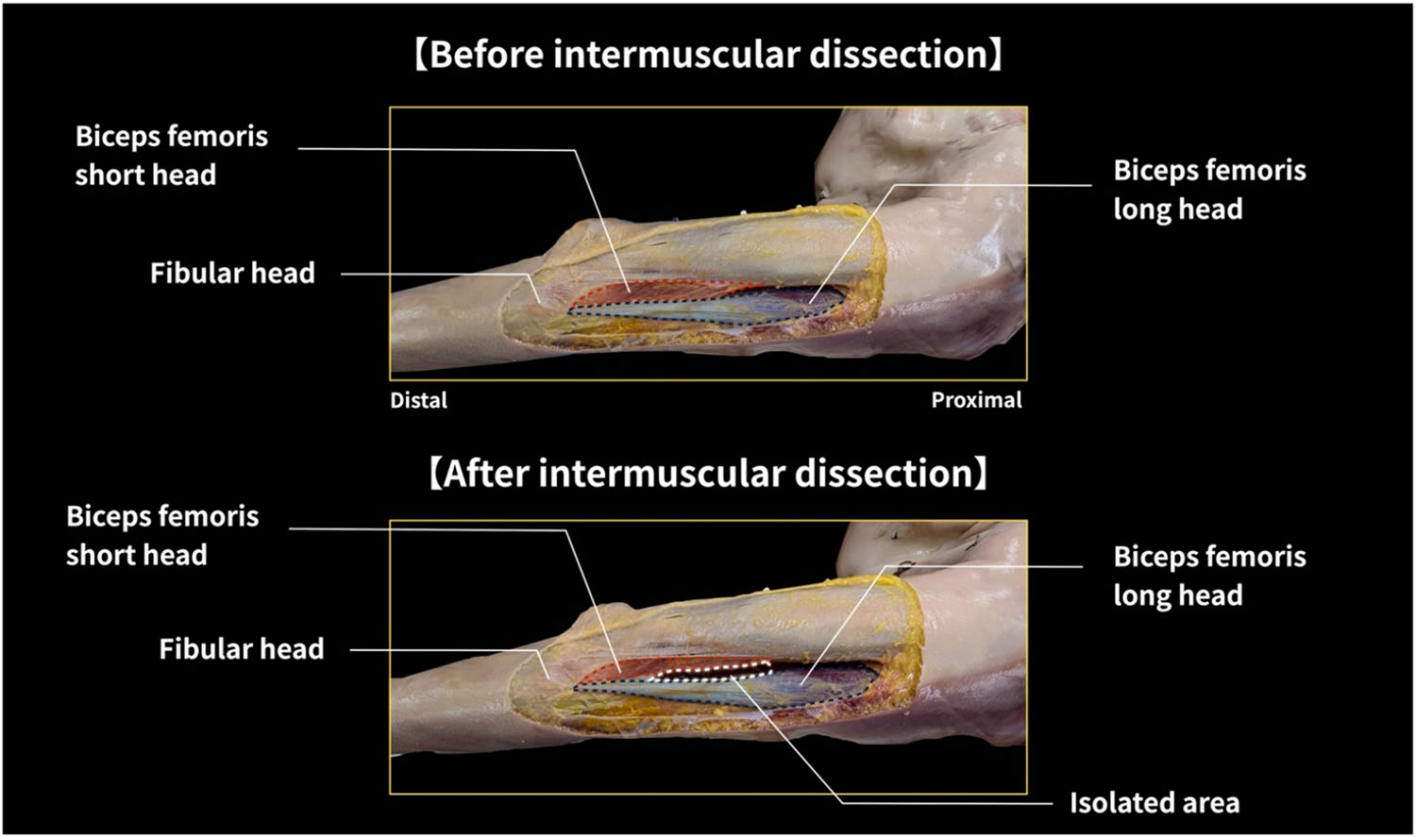
Representative images before and after separation of the intermuscular connection between the biceps femoris long head and short head. The muscle fibers of the BFsh are attached to the distal tendon of the BFlh. The attachment site was identified, and care was taken to preserve the integrity of the muscle fibers during dissection. The white dotted line indicates a representative region where intermuscular connective tissues were dissected between the BFlh and BFsh, excluding areas of direct muscle–tendon attachment. The extent of dissection varied slightly between specimens. The fibular head is marked to indicate the distal anatomical reference point. BFlh, biceps femoris long head; BFsh, biceps femoris short head.

This study was an extension of our previous work that examined shear modulus under intact and detached conditions (Nakao, Yamagata, et al., 2025). Therefore, this experiment was based on a previously established protocol, with the addition of a new condition involving dissection of intermuscular connections (Nakao, Yamagata, et al., 2025). Shear modulus measurements of the BFlh and BFsh were conducted under four distinct tissue conditions: (1) intact tissue; (2) removal of all layers from the skin to the deep fascia; (3) intermuscular dissection (Figure 1); and (4) detachment of the BFsh. For each condition, assessments were carried out across four joint configurations combining hip (0° or 90°) and knee (0° or 90°) angles. Joint angles were set using a goniometer and maintained at specific positions. During the entire experimental procedure, the muscles were kept immersed in Thiel solution at 22°C and 40% humidity to preserve tissue integrity and avoid changes resulting from environmental conditions such as temperature fluctuations, moisture loss, or drying. The study protocol was approved by the relevant ethics committee (approval number: 4‐1‐70).

### 2.2 Human cadavers

The individuals and their families consented to participate in the body donation program (Shiragiku‐kai) run by Sapporo Medical University for medical and scientific research. This study used eight legs from five human cadavers (five females; mean age at death, 83.2 ± 7.4 years; reported antemortem height, 153.4 ± 5.8 cm; reported antemortem weight, 44.1 ± 5.5 kg). Lower limbs were preserved using the Thiel embalming technique, an anatomical fixation method employing a mixture of propylene glycol and formalin (Thiel, 1992). This preservation technique is characterized by a low formalin concentration, which prevents excessive tissue hardening and maintains tissue flexibility (Benkhadra et al., 2011). Furthermore, Thiel‐embalmed specimens exhibit biomechanical properties, including elastic modulus and tensile strength, that are comparable to those of fresh skeletal muscles and tendons. Beger et al. reported that muscle stiffness and ultimate tensile strength in Thiel‐preserved specimens were not significantly different from those in fresh‐frozen samples (Beger et al., 2020). Similarly, Hohmanna et al. found that the elastic modulus of tendons preserved using the Thiel method closely approximated that of fresh tissue (Hohmann et al., 2019). Therefore, the mechanical properties of the hamstring muscles examined in this study were considered representative of living tissues.

### 2.3 Tissue processing

Tissue preparation was conducted in four sequential stages (Figure 1): (1) intact; (2) removal of tissues from the skin to the deep fascia; (3) separation of intermuscular connective tissues; and (4) BFsh detachment. After obtaining intact measurements, a skin incision was made from the lateral mid‐thigh to the distal thigh. Because selective separation of the BFsh was technically challenging, all specimens underwent stepwise dissection—first removing skin to deep fascia, then performing intermuscular dissection, and finally detaching the BFsh. Following the initial measurements in the intact condition, a longitudinal incision was made from the lateral mid‐thigh to its distal end.

**Figure 2:**
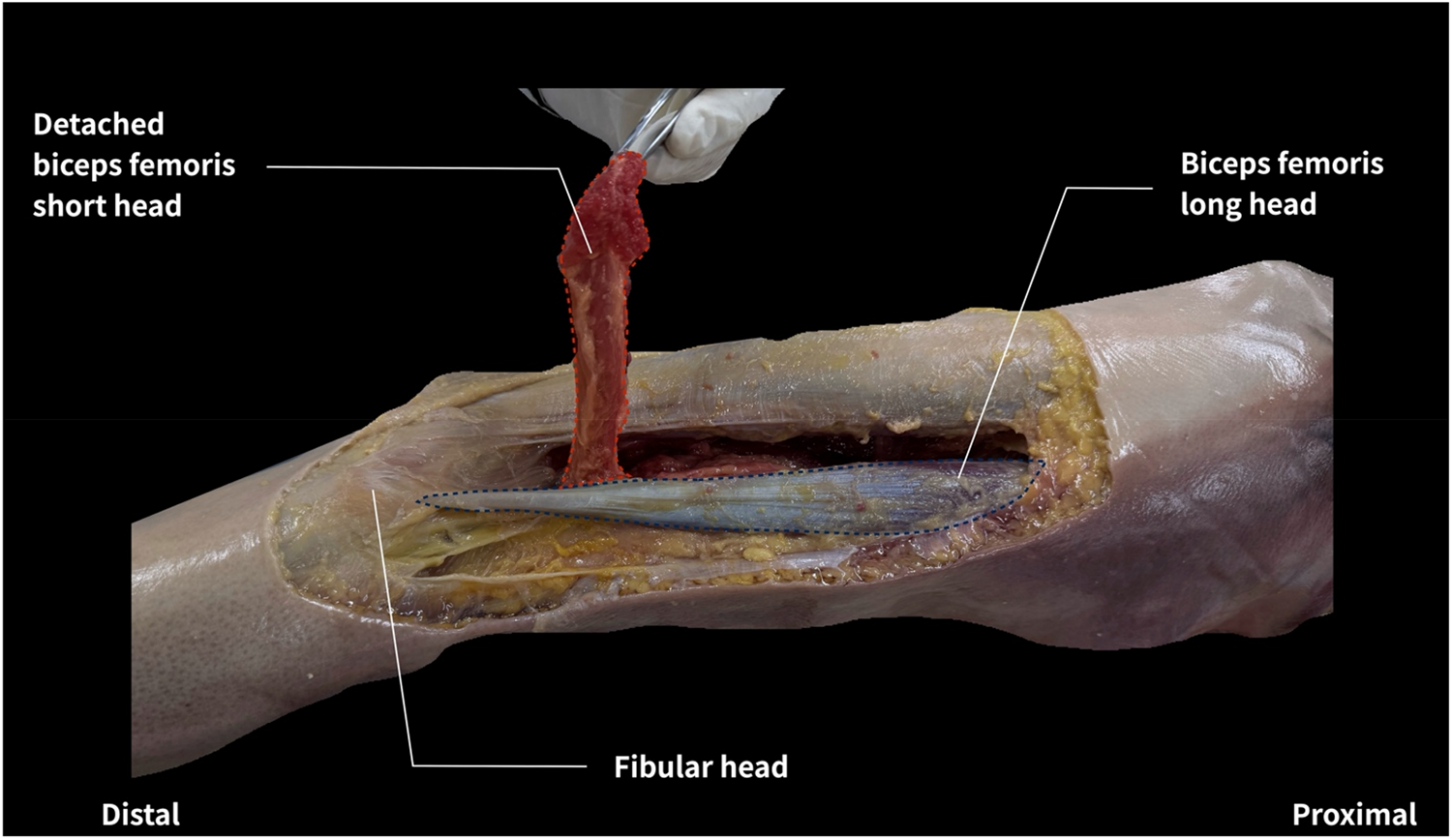
Complete detachment of the biceps femoris short head origin from the femur and lateral intermuscular septum. A representative image showing the BFsh being lifted with forceps after complete detachment from its origin. The dissection included the full extent of the BFsh origin, encompassing the posterior surface of the femur and the lateral intermuscular septum. The distal tendon remained intact. This procedure ensured removal of all proximal connective tissue contributions to the BFsh and minimized variability in tension transmission from residual attachment structures. BFsh, biceps femoris short head.

During the fascia removal step, the posterior thigh skin and subcutaneous fat were dissected to reveal the deep fascia, which was then incised along the same line to expose the BFlh and BFsh. For the intermuscular dissection condition, the connective tissues between the BFlh and BFsh were carefully dissected, except for the regions where the muscle fibers were attached to the tendon. This ensured that all the intermuscular connections between the BFsh and BFlh, apart from their tendinous attachments, were separated. The dissection followed the visible boundary between the two muscle bellies, typically extending from the distal third of the thigh to near the mid‐thigh region. However, precise measurements of the dissection extent (e.g., distance from the fibular head or percentage of BFlh length) were not recorded. Although individual variability was noted in the location and density of connective tissue, detailed morphometric evaluation was not performed. Importantly, in all specimens, the muscle fascicles and tendons of the BFlh and BFsh remained structurally intact throughout the dissection process. In the BFsh detachment condition, the entire origin of the BFsh—including its attachment to the posterior surface of the femur and the lateral intermuscular septum—was carefully detached, whereas the distal tendon at its insertion site was left intact. As shown in Figure 2, all associated tissue, including the lateral intermuscular septum, was removed.

### 2.4 Shear modulus measurement

Shear modulus was assessed via SWE using a linear ultrasound probe (Aixplorer version 12, MSK mode; Hologic, Marlborough, MA, USA) operating at 4–15 MHz. Young's modulus values ranged from 0 to 400 kPa, with blue and red indicating the lowest and highest values, respectively, on the color scale. Measurement locations were selected using B‐mode transverse ultrasound images, targeting a clear fascicle at a point approximately 30% distal along the line from the ischial tuberosity (100%) to the fibular head (0%). Once the BFlh and BFsh measurement points were identified, sewing push‐pin needles were placed as reference landmarks on the anterior and lateral thigh, enabling acquisition of long‐axis images at the corresponding sites. During imaging, the transducer was oriented along the muscle's longitudinal axis—specifically, along the aponeurosis for the BFlh and along the muscle fibers for the BFsh—to ensure alignment consistency. Each condition was recorded twice to improve measurement reliability.

### 2.5 Data analysis

**Figure 3:**
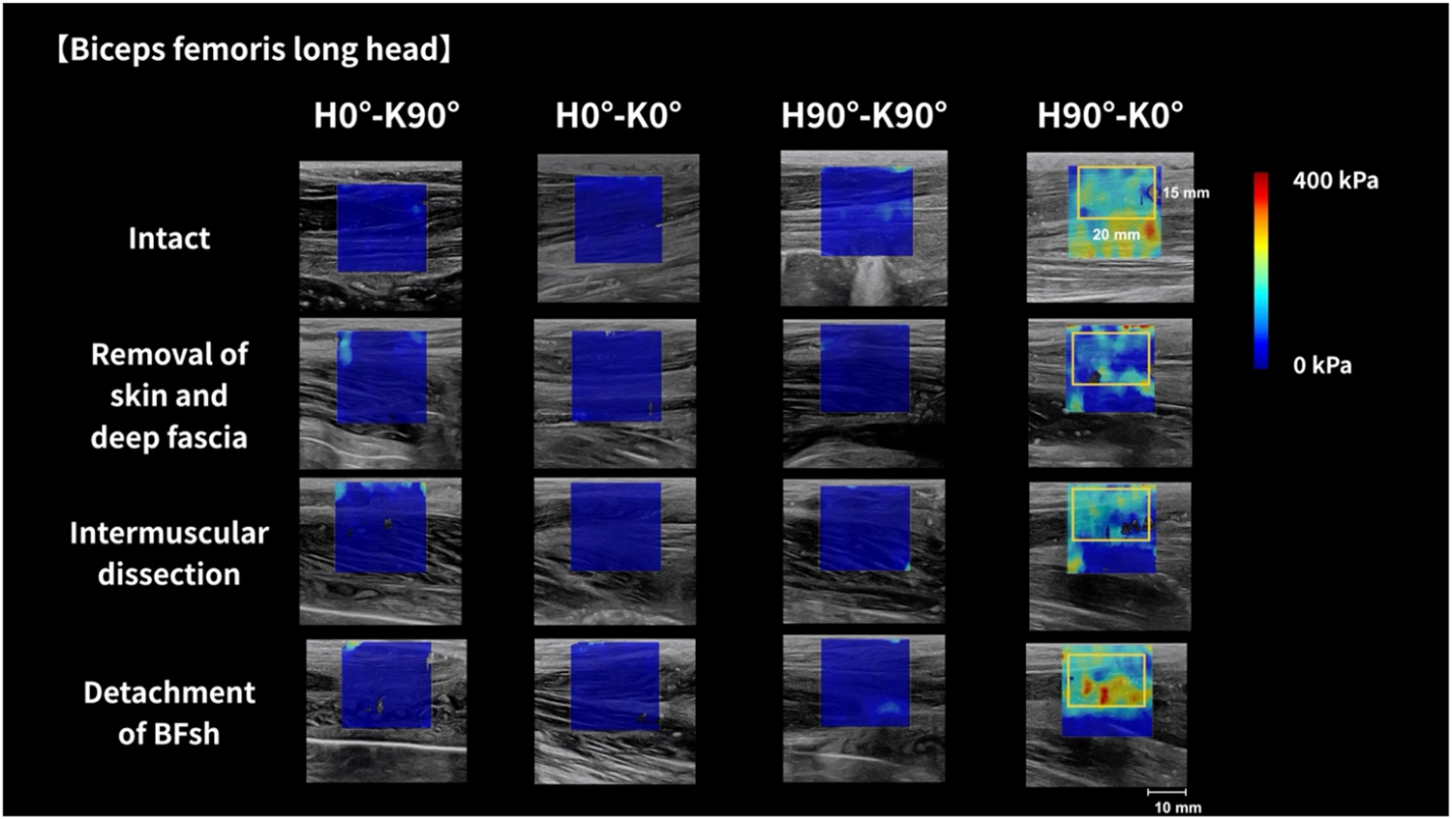
Typical shear modulus images of the biceps femoris long head for each limb position and tissue processing condition. The shear modulus was measured at hip (H0°, H90°) and knee (K0°, K90°) angles for each of the four tissue processing conditions (intact, removal of all tissues from the skin to the deep fascia, intermuscular dissection, and detachment of the BFsh). Colored areas represent the shear modulus map according to the scale shown on the right‐hand side of the figure. The rectangle indicates the region of interest used to determine the shear modulus. The upper limit of the color scale for the BFsh was 400 kPa. BFsh, biceps femoris short head.

**Figure 4:**
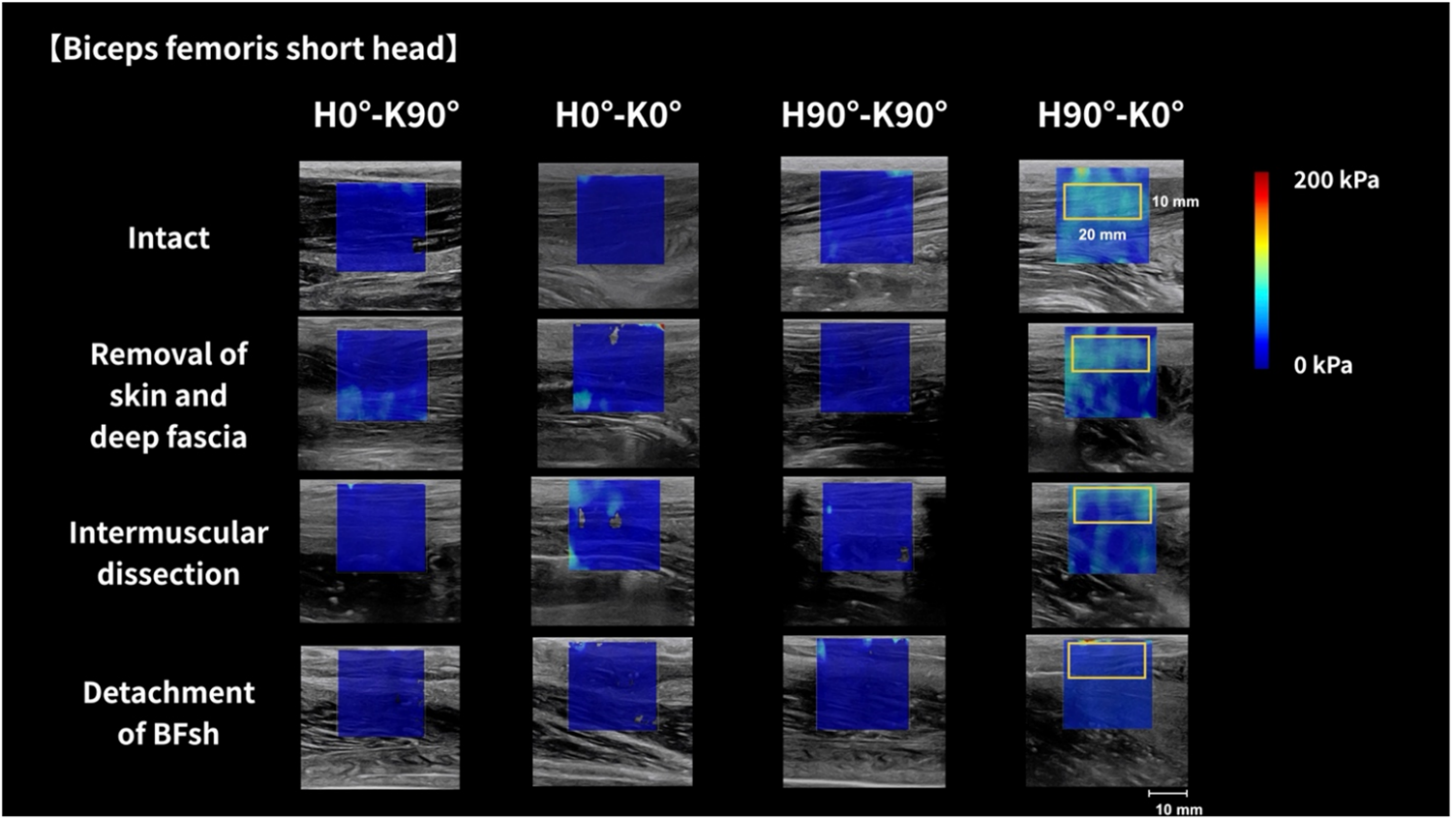
Typical shear modulus images of the biceps femoris short head for each limb position and tissue processing condition. The shear modulus was measured at hip (H0°, H90°) and knee (K0°, K90°) angles under each of the four tissue processing conditions (intact, removal of all tissues from the skin to the deep fascia, intermuscular dissection, and detachment of the BFsh). The upper limit of the color scale for the BFsh was 200 kPa. BFsh, biceps femoris short head.

JPEG‐format shear modulus images were analyzed using proprietary software (S‐14133 version 1.2; Takei Scientific Instrument Co. Ltd., Niigata, Japan) (Nakao et al., 2024). This software allows flexible adjustment of the region of interest (ROI) in terms of size and placement. A rectangular ROI was positioned to capture multiple muscle fascicles, and shear modulus was calculated using a color‐coded map. For the BFlh, the ROI was 20 mm wide and 15 mm high, with a color scale maximum of 400 kPa (Figure 3). For the BFsh, the ROI was 20 × 10 mm, with the upper limit set to 200 kPa (Figure 4). The center of the ROI was aligned with the midpoint of muscle thickness. Within the software, Young's modulus was estimated in kilopascals based on shear wave speed (c) using the formula E = 3ρc^2^, where ρ is the tissue density (assumed to be 1000 kg/m^3^). Since this method assumes isotropy in tissue, which muscle does not fully exhibit (Royer et al., 2011), shear modulus was obtained by dividing Young's modulus by 3. For analysis, the mean of the two shear modulus values per condition was used.

All statistical procedures were conducted using SPSS Statistics version 28.0 (IBM Corp., Armonk, NY, USA). Normality of the data was assessed with the Shapiro–Wilk test, which confirmed a normal distribution. Homogeneity of variances was verified with Levene's test. A three‐way repeated‐measures ANOVA was applied to analyze shear modulus variation, considering the muscle (BFlh vs. BFsh), tissue processing condition (intact, skin‐to‐fascia removal, intermuscular dissection, BFsh detachment), and joint position (H0°‐K90°, H0°‐K0°, H90°‐K90°, H90°‐K0°) as within‐subject factors. When significant main effects were found, Bonferroni‐adjusted post hoc tests were conducted. A significance threshold of 5% was adopted, and partial eta squared was used to report effect sizes.

## 3 RESULTS

**Figure 5:**
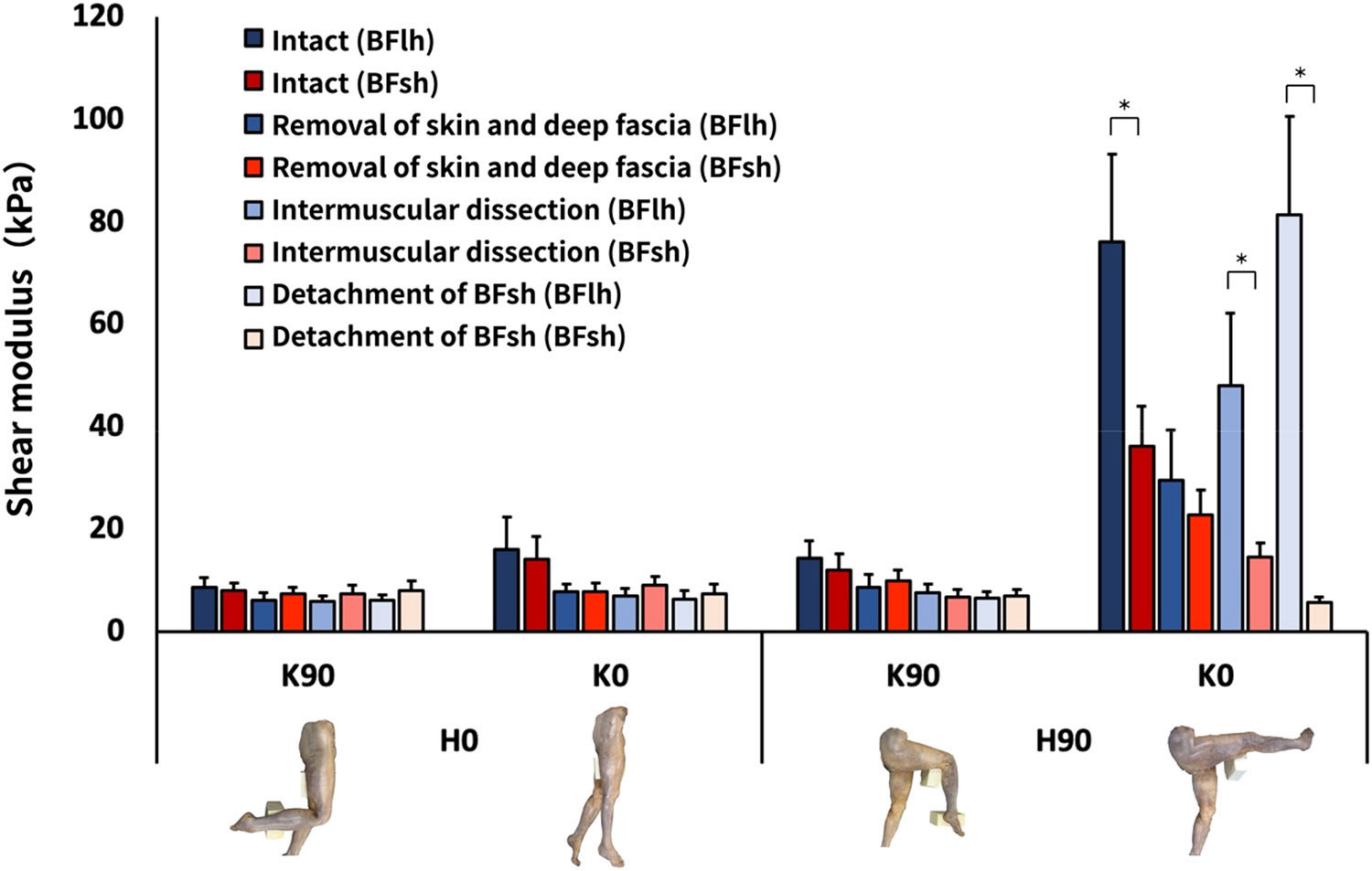
The shear modulus of the biceps femoris long head and short head under different limb positions and treatment conditions. Values are shown as means ± standard errors. Multiple comparisons revealed a significant difference between the muscles only under the H90°–K0° condition. In the intact condition, the shear modulus of the BFlh was significantly higher than that of the BFsh. After skin and fascia removal, no significant differences were observed. However, following intermuscular dissection and BFsh detachment, the shear modulus of the BFlh was significantly higher than that of the BFsh. *p < 0.05 versus BFsh. BFlh, biceps femoris long head; BFsh, biceps femoris short head.

**Figure 6:**
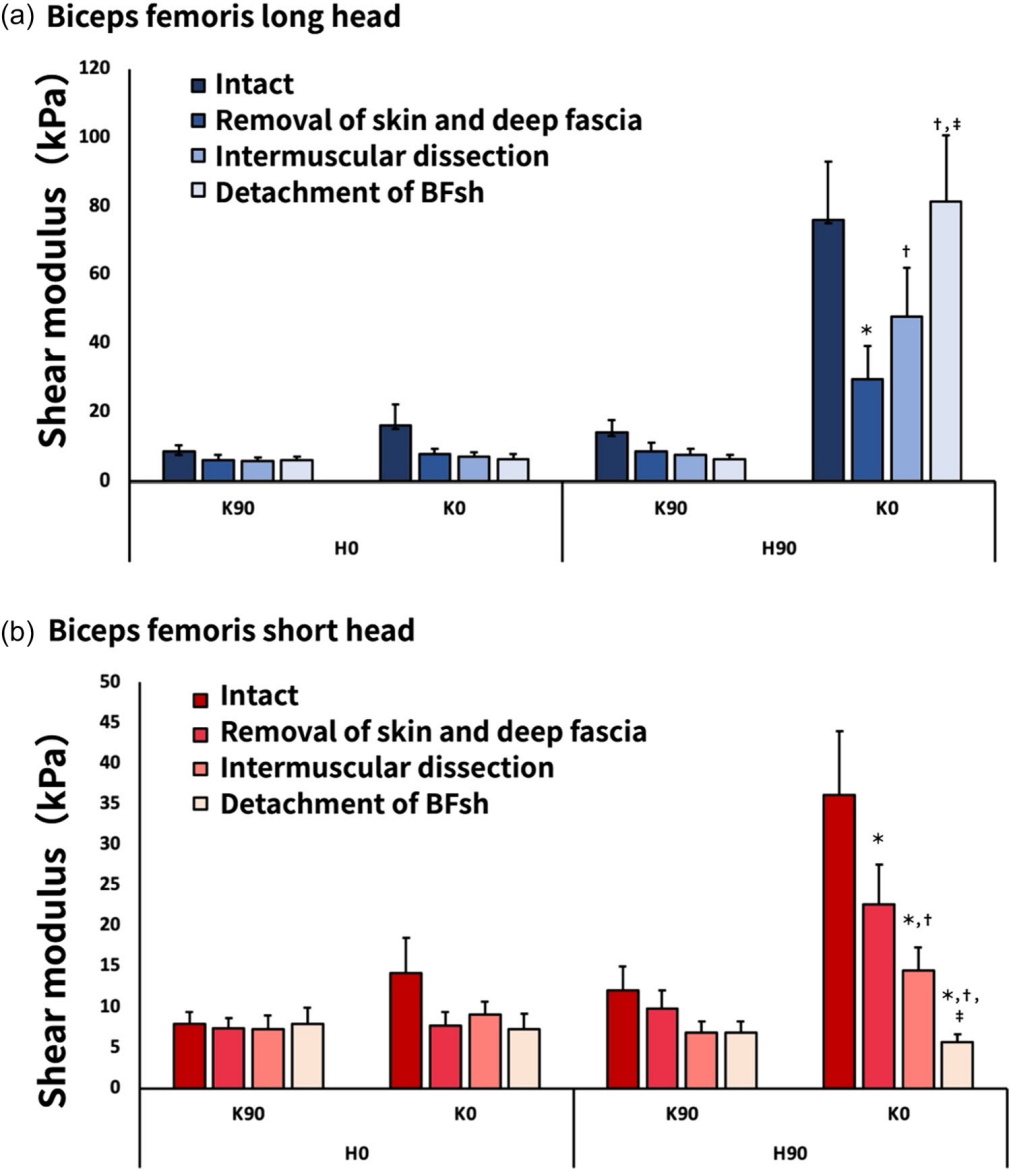
Mean shear modulus of the biceps femoris long head (a) and short head (b) under different treatment conditions for each limb position. Values are shown as means ± standard errors. For the BFlh, multiple comparisons showed a significant decrease in shear modulus after skin and fascia removal, followed by a significant increase after intermuscular dissection and BFsh detachment. For the BFsh, shear modulus significantly decreased after each successive tissue processing step. *p < 0.05 versus intact, ^†^ *p* < 0.05 versus skin and fascia removal, ^‡^ *p* < 0.05 versus intermuscular dissection. BFlh, biceps femoris long head; BFsh, biceps femoris short head.

**Figure 7:**
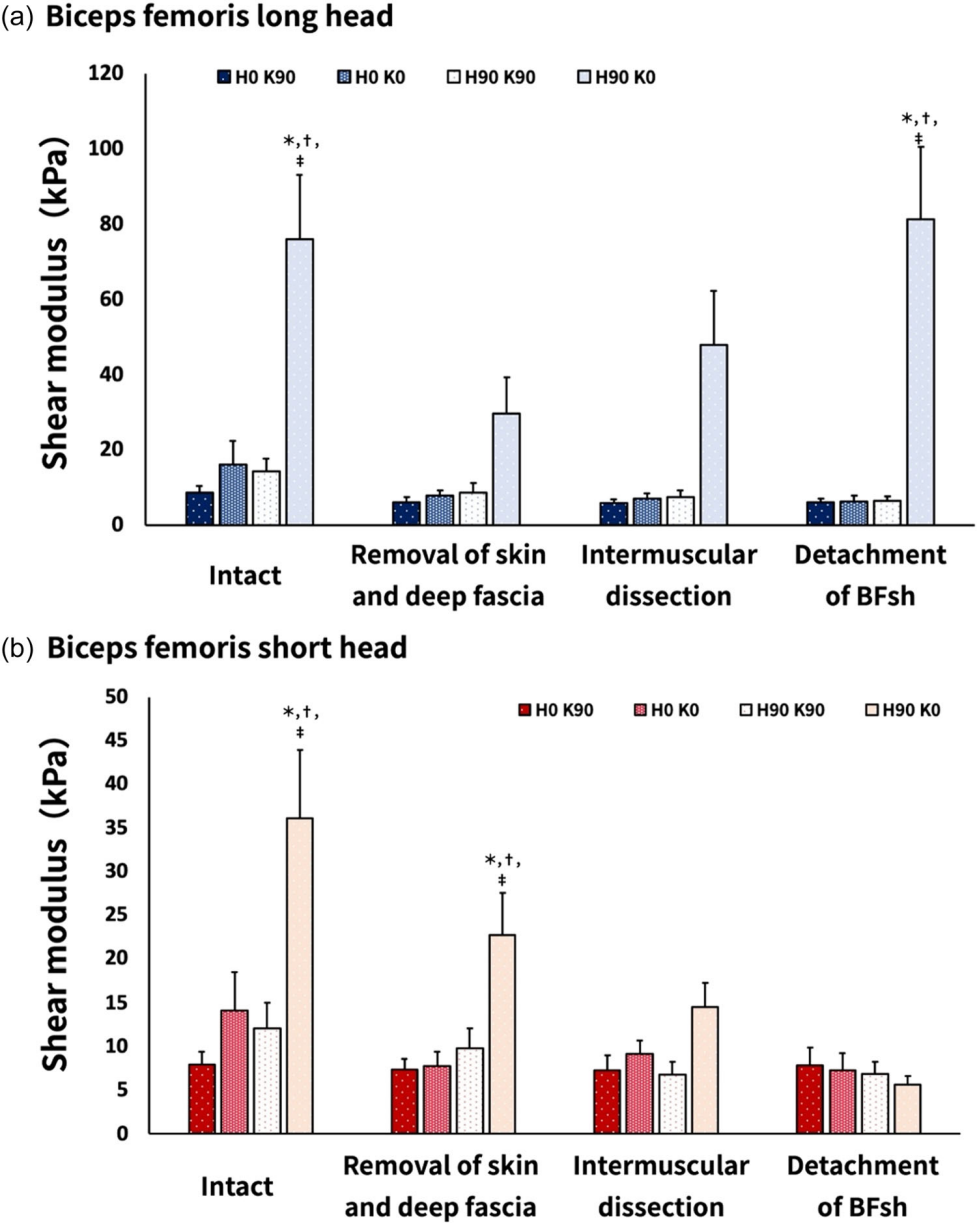
Mean shear modulus of the biceps femoris long head (a) and short head (b) under different limb positions for each treatment condition. Values are shown as means ± standard errors. For the BFlh, before tissue processing and after skin and fascia removal, the shear modulus was significantly higher at the H90°–K0° position compared to other angles. For the BFsh, a significantly higher shear modulus at the H90°–K0° position was observed before tissue processing and after BFsh detachment. *p < 0.05 versus H0°–K90°, ^†^ *p* < 0.05 versus H0°–K0°, ^‡^ *p* < 0.05 versus H90°–K90°. BFlh, biceps femoris long head; BFsh, biceps femoris short head.

Three‐way ANOVA revealed the following significant interactions: muscle × tissue processing × position (F_9,63_ = 8.351, *p* < 0.001, ηp^2^ = 0.544); muscle × tissue processing (F_3,21_ = 10.175, *p* < 0.001, ηp^2^ = 0.592); muscle × position (F_3,21_ = 16.577, *p* < 0.001, ηp^2^ = 0.703); and tissue processing × position (F_9,63_ = 9.958, *p* < 0.001, ηp^2^ = 0.587) (Figures 5, 6, 7).

Regarding comparisons between the biceps femoris muscles, the shear modulus of the BFlh was significantly higher than that of the BFsh at H90° and K0° before tissue processing (*p* < 0.01); however, no difference was observed between the muscles after removal of all tissues from the skin to the deep fascia (*p* = 0.09) (Figure 5). After intermuscular dissection and BFsh detachment, the shear modulus of the BFlh was again significantly higher than that of the BFsh (*p *< 0.01) (Figure 5).

Regarding the relationship between tissue treatment and position, the shear modulus of the BFlh was significantly decreased at H90°–K0° after removal of all tissues from the skin to the deep fascia compared with the intact condition (*p *< 0.01) (Figure 6a). In contrast, the shear modulus of the BFlh significantly increased after intermuscular dissection and BFsh detachment (*p* < 0.01). The shear modulus of the BFsh decreased progressively following the removal of all tissues from the skin to the deep fascia, intermuscular dissection, and BFsh detachment compared with the intact condition (*p *< 0.01) (Figure 6b).

Regarding the effect of position, the shear modulus of the BFlh was significantly higher at H90°–K0° in the intact and BFsh detachment conditions compared with the other positions (*p *< 0.01) (Figure 7a). However, no significant differences were observed at this position after removal of all tissues to the deep fascia or after intermuscular dissection. The shear modulus of the BFsh was significantly higher at H90°–K0° in the intact condition and after removal of tissues from the skin to the deep fascia compared with other positions (*p *< 0.01) (Figure 7b). In contrast, intermuscular dissection and BFsh detachment showed no significantly differences between positions (Figure 7b).

## 4 DISCUSSION

This study investigated the mechanical interaction between the BFlh and BFsh by examining the effects of intermuscular connection dissection and BFsh detachment on muscle shear modulus. The key findings were that the shear modulus of the BFlh increased by 62.2%, whereas that of the BFsh decreased by 36.0% after dissection of the intermuscular connections, indicating a redistribution of mechanical load between the two heads. These results provide new insights into multidirectional force transmission within the biceps femoris and highlight the role of intermuscular connections in modulating muscle tension.

Although previous studies have primarily emphasized force transmission along the longitudinal axis of the muscle–tendon unit (Nakao, Yamagata, et al., 2025), our findings suggest that the BFlh and BFsh engage in integrated multidirectional mechanical interactions. From a vector decomposition perspective, tensile forces within a muscle can be divided into vertical (longitudinal) and horizontal (transverse or oblique) components relative to the muscle's principal axis (Farfán et al., 2021), and force transmission during elongation occurs not only along the vertical axis but also in the horizontal direction (Finni et al., 2023). In the intact condition, intermuscular connections may help redistribute part of the transverse stress toward the BFsh, potentially reducing the net mechanical burden on the BFlh. After dissection, this redistribution appears to diminish, increasing the mechanical load borne by the BFlh. Rather than interpreting this as a strict directional shift of force from the BFsh to the BFlh, it may be more accurate to view it as a loss of mechanical cooperation between the two heads, resulting in load concentration within the BFlh. Therefore, the mechanical behavior of the biceps femoris complex should be understood as the result of integrated multidirectional interactions rather than isolated uniaxial force transmission.

These findings have important clinical implications for understanding hamstring injury mechanisms. The biceps femoris, particularly the musculotendinous T‐junction, is the most frequently injured hamstring muscle (Entwisle et al., 2017; Shamji et al., 2021). Although this study did not directly assess the T‐junction, the observed redistribution of mechanical load following intermuscular dissection suggests that disruption of intermuscular connectivity can alter intramuscular force transmission pathways. Such alterations may indirectly influence the mechanical loading near the T‐junction and contribute to injury susceptibility in regions exposed to high tensile stress. To mitigate such risks, enhancing the mechanical contribution of the BFsh—by increasing its muscle volume and improving neuromuscular activation—may help distribute the tensile load more effectively within the biceps femoris. Eccentric knee flexion exercises, such as the Nordic hamstring exercise and leg curls, preferentially activate the BFsh and semitendinosus (Bourne, Duhig et al., 2017; Bourne, Williams et al. 2017; Bourne et al., 2018). Repeated activation of the BFsh through these exercises may improve its capacity to generate tension and assist in load sharing with the BFlh. Consequently, BFsh strengthening may reduce the excessive mechanical load placed on the BFlh, particularly during high‐demand movements. This improved intermuscular load distribution may help lower the risk of injury recurrence, particularly in athletes with a history of hamstring strain (Mendiguchia et al., 2013).

In addition to mechanical interactions, the functional relationship between the BFlh and BFsh is also modulated by their distinct neural innervations. The BFlh is innervated by the tibial branch of the sciatic nerve, similar to other posterior thigh muscles and the gastrocnemius, whereas the BFsh receives input from the common peroneal nerve, which also supplies anterior lower leg muscles such as the tibialis anterior (Woodley & Mercer, 2005). This separation in motor control suggests that the two heads may not always be co‐activated, especially during complex or high‐speed movements. From a clinical perspective, this differential innervation could influence neuromuscular coordination and adaptation following injury, particularly in rehabilitation settings aimed at optimizing intermuscular force sharing. Although cadaveric experiments cannot capture neural influences, future in vivo studies incorporating electromyography or nerve stimulation may provide further insights into how neural pathways contribute to the functional integration of the biceps femoris heads.

This study has some limitations. First, following previous studies, shear modulus was measured at specific, fixed joint angles (H0°, H90° for the hip and K0°, K90° for the knee) (Nakao, Yamagata, et al., 2025). Although this method provides controlled and reproducible measurements, it does not reflect the full range of motion or dynamic activities experienced by the muscles in real‐life scenarios (Kellis & Blazevich, 2022). Second, although vector decomposition offers a theoretical framework for understanding force transmission, direct in vivo measurements of force vectors have not yet been performed. Future studies should incorporate in vivo imaging and biomechanical modeling to validate these findings and further investigate the implications of altered force transmission on hamstring injury risk and muscle function. Third, this study did not assess muscle morphology variables such as fascicle length, cross‐sectional area, and pennation angle, which are important for analyzing the BFsh and other muscle components. Since mechanical parameters are influenced by muscle morphology (Nakao et al., 2023; Nakao et al., 2024; Nakao, Kodesho, et al., 2025), future studies should include simultaneous measurements of these morphological characteristics to enable a more detailed analysis. Fourth, shear modulus was measured at only a single location within each muscle. Given that regional intramuscular differences in stiffness can exist (Miyamoto et al., 2020), measuring a single region may not fully capture the mechanical heterogeneity of the muscle. Fifth, although intermuscular dissection was a central component of this study, we did not quantify the extent of dissection in terms of the distance from the fibular head or as a proportion of the full BFlh length from the ischial tuberosity to the fibular head. Although dissection was consistently performed from the distal to the mid‐thigh region across all specimens, individual variability in the location and density of intermuscular connective tissue was observed. However, we did not conduct detailed morphometric analyses to quantify these variations. Future studies should include precise anatomical measurements and morphological assessments to better characterize the structural heterogeneity of the intermuscular connections. Finally, all cadavers were obtained from older individuals. A previous study reported that older subjects exhibited significantly higher muscle tension than younger subjects (Pavan et al., 2020). It is also widely recognized that advanced age is a strong risk factor for hamstring muscle strain (Green et al., 2020). Based on these findings, age‐related changes in muscle and connective tissue properties may have influenced the magnitude and pattern of force transmission observed in this study. Therefore, caution is warranted when generalizing the present findings to younger populations or athletic individuals. Future studies including younger or more diverse samples are needed to better understand age‐related differences in hamstring muscle mechanics and their implications for injury risk.

## 5 CONCLUSION

The intermuscular connections between the BFlh and BFsh play a crucial role in force transmission and mechanical load sharing within the biceps femoris muscle. The observed increase in BFlh shear modulus and decrease in BFsh shear modulus following intermuscular dissection suggest that these anatomical connections contribute to the distribution of mechanical loads across the muscle heads.

## AUTHOR CONTRIBUTIONS

**Gakuto Nakao:** Conceptualization; methodology; formal analysis; investigation; writing—original draft; visualization. **Norio Hayashi:** Conceptualization. **Jurdan Mendiguchia:** Conceptualization. **Raki Kawama:** Conceptualization. **Ginji Nara:** Data curation; investigation. **Risa Adachi:** Data curation; investigation. **Koki Ishiyama:** Data curation; investigation. **Kazuyoshi Kozawa:** Data curation; investigation. **Keita Sekiguchi:** Data curation; investigation. **Kanna Nagaishi:** Writing—review and editing; validation. **Kousuke Shiwaku:** Writing—review and editing; validation. **Nobuhiro Aoki:** Writing—review and editing; validation. **Masaki Katayose:** Writing—review and editing; validation. **Keigo Taniguchi:** Writing—review and editing; supervision; project administration; resources. All authors reviewed and approved the final version of the manuscript and agree to be accountable for all aspects of the work, ensuring that questions related to the accuracy or integrity of any part of the study are appropriately investigated and resolved.

## CONFLICT OF INTEREST STATEMENT

The authors declare no conflicts of interest.

## Data Availability

The data reported in this study are available upon request from the corresponding author.
